# Activation of Sigma Receptors With Afobazole Modulates Microglial, but Not Neuronal, Apoptotic Gene Expression in Response to Long-Term Ischemia Exposure

**DOI:** 10.3389/fnins.2019.00414

**Published:** 2019-05-15

**Authors:** Adam A. Behensky, Christopher Katnik, Huquan Yin, Javier Cuevas

**Affiliations:** Department of Molecular Pharmacology and Physiology, College of Medicine, University of South Florida, Tampa, FL, United States

**Keywords:** afobazole, ischemia, apoptosis, Bax, caspase-3, Bcl-2

## Abstract

Stroke continues to be a leading cause of death and serious long-term disability. The lack of therapeutic options for treating stroke at delayed time points (≥6 h post-stroke) remains a challenge. The sigma receptor agonist, afobazole, an anxiolytic used clinically in Russia, has been shown to reduce neuronal and glial cell injury following ischemia and acidosis; both of which have been shown to play important roles following an ischemic stroke. However, the mechanism(s) responsible for this cytoprotection remain unknown. Experiments were carried out on isolated microglia from neonatal rats and cortical neurons from embryonic rats to gain further insight into these mechanisms. Prolonged exposure to *in vitro* ischemia resulted in microglial cell death, which was associated with increased expression of the pro-apoptotic protein, Bax, the death protease, caspase-3, and reduced expression in the anti-apoptotic protein Bcl-2. Incubation of cells with afobazole during ischemia decreased the number of microglia expressing both Bax and caspase-3, and increased cells expressing Bcl-2, which resulted in a concomitant enhancement in cell survival. In similar experiments, incubation of neurons under *in vitro* ischemic conditions resulted in higher expression of Bax and caspase-3, while at the same time expression of Bcl-2 was decreased. However, unlike observations made in microglial cells, afobazole was unable to modulate the expression of these apoptotic proteins, but a reduction in neuronal death was still noted. The functional state of surviving neurons was assessed by measuring metabolic activity, resting membrane potential, and responses to membrane depolarizations. Results showed that these neurons maintained membrane potential but had low metabolic activity and were unresponsive to membrane depolarizations. However, while these neurons were not fully functional, there was significant protection by afobazole against long-term ischemia-induced cell death. Thus, the effects of sigma receptor activation on microglial and neuronal responses to ischemia differ significantly.

## Introduction

Ischemic stroke has been shown to be a multicellular disease, and any attempts to treat this disease must target several cell types (Hall et al., 2009b; Leonardo et al., 2010; Cuevas et al., 2011a,b). Neurons have previously been the primary target in the treatment of stroke, but microglia and astrocytes play a dynamic role in the pathophysiology of stroke (Hu et al., 2012; Katnik et al., 2016; Xu et al., 2017). In response to stroke or other types of CNS injury, microglia become activated and migrate to the site of injury (Kierdorf and Prinz, 2013). While the initial action of these cells appears neuroprotective in nature, overtime activated microglial cells transform to a phenotype that promotes neurodegeneration (Hu et al., 2012; Hellwig et al., 2013). It has been proposed that microglia play a major role in the progression of neuronal injury days and weeks after an ischemic stroke (Hu et al., 2012; Hellwig et al., 2013). Conversely, microglial cells have also been shown to provide trophic support to neurons and can promote enhanced neuronal survival and decreased stroke injury (Kitamura et al., 2004). Therefore, it is important to identify therapeutic treatments that reduce neuronal death and prevent microglia death while preserving these cells in a beneficial phenotype.

Sigma (σ) receptors play a vital role in neuroprotection and cell regulation (Ajmo et al., 2006; Katnik et al., 2006; Su et al., 2010). The σ-1 receptor is an inter-organelle chaperone localized to the mitochondrion-associated ER membrane (MAM) (Su et al., 2010). This σ-1 receptor-MAM complex plays a major role in cell signaling, mitochondrial function, maintaining intracellular calcium homeostasis and the expression of cellular proteins (Tsai et al., 2009). In contrast, the function of σ-2 receptors is less understood, in part because the gene encoding this receptor was only recently cloned (Alon et al., 2017). σ-2 receptors are important in regulating intracellular calcium (Vilner and Bowen, 2000) and in the activation and migration of microglial cells (Cuevas et al., 2011b; Iniguez et al., 2013). Largely distributed throughout the mammalian body, σ receptors perform a number of other functions such as regulating membrane ion channels (Tchedre et al., 2008; Hayashi et al., 2000), altering expression of anti-apoptotic and pro-apoptotic transcription factors (Yang et al., 2007; Tchedre and Yorio, 2008; Meunier and Hayashi, 2010) and modulating activation and migration of microglial cells (Hall et al., 2009a). Additionally, these receptors have been shown to provide protection to both neurons and glial cells following *in vitro* ischemia and *in vivo* middle cerebral artery occlusion (MCAO) stroke models (Ajmo et al., 2006; Katnik et al., 2006, 2016). However, the cellular mechanisms responsible for the reduced cell death remain poorly understood and it is unknown if activation of σ receptors provides cytoprotection in neurons and microglial cells via identical pathways.

Our laboratory has previously shown the pan-selective sigma receptor agonist afobazole (5-ethoxy-2-[2-(morpholino)-ethylthio]benzimidazole), an anxiolytic used clinically in Russia, to be protective in neurons (Cuevas et al., 2011a) and microglia (Cuevas et al., 2011b) during *in vitro* ischemia and acidosis. Furthermore, afobazole prevented neuronal and glial cell death following MCAO, an *in vivo* rat ischemic stroke model (Katnik et al., 2014). Other studies have suggested that afobazole may provide neuroprotection in a variety of pathophysiological states involving oxidative stress and glutamate toxicity (Zenina et al., 2005). Recently, our laboratory showed that afobazole maintained neuronal and microglial cell viability when exposed to amyloid-β_25-35_ by downregulating the pro-apoptotic protein Bax and the death protease caspase-3 while preserving Bcl-2 expression (Behensky et al., 2013b,c). It is of significant interest to determine if similar mechanisms are involved in σ receptor-mediated protection against ischemic injury and death.

Experiments were carried out to determine how afobazole affects neuronal and microglial responses to prolonged *in vitro* ischemia in rat cortical cells. Afobazole reduced ischemia-induced cell death in both microglia and neurons. In addition, afobazole decreased cell death by modulating the upregulation of Bax and caspase-3 following ischemia in microglia but not in neurons. Moreover, afobazole preserved Bcl-2 expression in microglia, but not neurons following exposure to 24 h ischemia. However, while the activation of σ receptors was able to confer longer neuronal viability against prolonged ischemic exposure, these neurons were shown to be unresponsive to depolarizations and exhibited low metabolic activity. Thus, activation of σ receptors reduces neuronal and glial cell death through distinct mechanisms.

## Materials and Methods

### Primary Cultures of Microglia

Primary cultures of microglia were prepared from Sprague-Dawley mixed sex rat pups (post-natal days 2–3) as previously described by our laboratory (Hall et al., 2009a; Cuevas et al., 2011b). The mixed glial cultures were incubated for 7–12 days at 37°C prior to experiments being carried out. Microglia were mechanically separated from the cultures by brief shaking. Isolated microglia were re-suspended in DMEM PLUS containing: 500 mL Dulbecco’s modified Eagle medium with 4.5 g/L glucose, L-glutamine and sodium pyruvate (DMEM) (Corning Cellgro, Manassas, VA, United States), 40 mL horse serum (Corning Cellgro), 12.5 mL heat-inactivated fetal bovine serum (Thermo Scientific Hyclone, Logan, UT, United States), and 5 mL 10X antibiotic/antimycotic (Corning Cellgro). These cells were plated on poly-L-lysine coated glass coverslips for 1 day prior to the addition of ATP or chemical ischemia.

### Membrane Morphology

Morphological changes to microglia induced by ATP were assessed by plating the cells on poly-L-lysine coated coverslips, serum starving the cells for 4 h in DMEM, and exposing the cells to 100 μM ATP for 10 min at 37°C. When afobazole or DTG (1,3-Di-o-tolylguanidine) were used (30 μM), microglia were incubated in the σ ligands (in DMEM) for 10 min prior to ATP exposure. Membrane ruffling was visualized using a 63X oil emersion objective and labeling the cells with the filamentous actin probe, phalloidin (AlexaFluor 488 conjugated, Life Technologies, Grand Island, NY, United States). Quantification of membrane ruffling was carried out as previously described (Hall et al., 2009a). Briefly, cell ruffling was scored as: “0” – no ruffling and multiple filopodia; “1” – ruffling and filopodia; “2” – fully ruffled with no filopodia.

### Primary Cultures of Cortical Neurons

All experiments were carried out on cultured cortical neurons from mixed-sex embryonic day 18 (E18) Sprague-Dawley rats (Harlan, Indianapolis, IN, United States). Methods used here were identical to those previously reported for the isolation and culturing of these cells (Katnik et al., 2006). Briefly, cortical neurons were plated on poly-L-lysine coated coverslips and cultured in B27 and L-glutamine supplemented Neurobasal medium (Neurobasal Complete) (Life Technologies, Grand Island, NY, United States). Cells were used for experiments between 10 and 21 days in culture, which permits synaptic contact formation and yields robust responses to other pathophysiological conditions such as *in vitro* ischemia and acidosis (Katnik et al., 2006; Herrera et al., 2008). Animals were cared for in accordance with the regulations and guidelines set forth by the University of South Florida’s College of Medicine Institution on Animal Care and Use Committee.

### *In vitro* Ischemia

In all experiments involving ischemia, the chemical ischemia model used previously in our laboratory was employed (Katnik et al., 2006; Mari et al., 2010; Cuevas et al., 2011a,b; Behensky et al., 2013a). Briefly, culture media was supplemented with 4 mM sodium azide to inhibit mitochondrial function and cells were incubated in this media for the indicated times.

### Cell Death Assay

Neurons or microglia were plated on poly-L-lysine coverslips and incubated at 37°C for 72 h in Neurobasal Complete or DMEM Plus, respectively, with 4 mM sodium azide in the absence (Control) and presence of 100 μM afobazole for neurons and 30 μM afobazole for microglia. Concentration of afobazole chosen for the two cell types are based on maximal effect reported for this compound in our previous studies in these respective cell types (Cuevas et al., 2011a,b; Behensky et al., 2013b,c). The coverslips were rinsed with warm PBS followed by 30 min incubation at room temperature in PBS with 4 μL of 2 mM ethidium homodimer-1 (EthD-1) dissolved in 1:4 DMSO:H_2_O solution. The coverslips were washed with PBS and deionized water, dried, and mounted on microscope slides with Vectashield Hardset mounting media (Vector Labs, Burlingame, CA, United States). EthD-1 loaded cells were illuminated with light at 530 nm and visualized at 645 nm using a Zeiss Axioskop 2 equipped with a 20X objective. EthD-1-positive cells were identified and counted using Image-J in four random fields per slide, and the average for the fields used as the value for the slide. For each experiment, a minimum of three slides from each preparation were used, and the results of at least three experiments were averaged together for each condition tested.

### Immunocytochemistry

Neurons or microglia plated on poly-L-lysine coverslips were incubated at 37°C for 24 h in Neurobasal Complete or DMEM Plus with 4 mM sodium azide in the absence and presence of 100 or 30 μM afobazole, respectively. The coverslips were rinsed with phosphate-buffered saline (PBS) followed by an ethanol step-wise fixation. The cells were permeabilized with 0.1% Triton X-100 in PBS for 15 min, then rehydrated with PBS and washed with 0.5% bovine serum albumin (BSA) in PBS. Blocking was achieved with 45 min incubation in 2% BSA, followed by multiple washes with 0.5% BSA. Primary antibodies were diluted in PBS with 0.5% BSA and applied onto cells at 4°C for 24 h. Primary antibody dilutions were as follows: Bax 1/20, Caspase-3 1/25, and Bcl-2 1/100. The cells were then washed multiple times with 0.5% BSA in PBS and then incubated in secondary antibodies for 60 min at room temperature. Secondary antibodies were either Alexa Fluor 488 conjugated anti-mouse or anti-rabbit IgG diluted at a ratio of 1/300 in PBS with 0.5% BSA. Following incubation in secondary antibodies, cells were washed with 0.5% BSA in PBS and then with PBS alone. Coverslips were rinsed with deionized water and inverted onto a slide coated with VectaShield containing DAPI. DAPI and the Alexa Fluor 488 conjugated secondary antibodies were illuminated at 359 and 485 nm and visualized at 461 and 530 nm, respectively, using a Zeiss Axioskop 2 with a 40X objective. Images of DAPI and Alexa Fluor 488 positive cells were counted and merged to demonstrate co-localization using Image-J software. Four random fields per slide were used to determine an average value for the slide. For each experiment, a minimum of three slides were used, and the results of at least three experiments were averaged together.

### Calcium Imaging

Intracellular Ca^2+^ concentrations ([Ca^2+^]_i_) were measured in isolated cortical neurons using ratiometric fluorometry as previously described (Katnik et al., 2006). Neurons were loaded with fura-2 by adding 3 μg/ml of the acetoxymethylester form of fura-2 (fura-2 AM) in 0.3% DMSO to the media and incubating the cells at room temperature for 1 h. Prior to beginning the experiments, the coverslips were rinsed with physiological saline solution (PSS) consisting of (in mM): 140 NaCl, 5.4 KCl, 25 HEPES, 20 glucose, 1.3 CaCl_2_, and 1.0 MgCl_2_ (pH to 7.4 with NaOH). For each cell, [Ca^2+^]_i_, calculated using the Grynkiewicz equation, was measured at 1 Hz for 100 s and average baseline, peak and net [Ca^2+^]_i_ were determined.

Data points for calcium imaging experiments represent peak and total means ± standard error of the mean (SEM). Data from immunocytochemical and cell death assays were analyzed using Image J followed by statistical analysis using SigmaPlot 11 software (Systat Software, Inc., San Jose, CA, United States).

### Electrophysiological Measurements

Neurons were isolated and plated on glass coverslips as described above. Cells were used after 10–21 days *in vitro*. Prior to use, cells were incubated in Neurobasal Complete with 4 mM Na-azide in the absence or presence of 100 μM afobazole for 24 h and then Neurobasal Complete for 72 h. Control cells were subjected to similar solution changes in the absence of azide and afobazole. Membrane potentials were recorded using the perforated, whole-cell patch clamp configuration as previously described (Cuevas et al., 1997). Briefly, glass coverslips plated with neurons were transferred to a recording chamber and continuously perfused with external solution at a rate of 350 μl/min. Patch electrodes were pulled from thin-walled borosilicate glass (World Precision Instruments, Inc., Sarasota, FL, United States) using a Sutter Instruments P-87 pipette puller (Novato, CA, United States) and had resistances of 2.0–3.0 MΩ. Electrical access was achieved with a pipette solution containing amphotericin B (Rae et al., 1991). An amphotericin B stock solution (60 mg/ml in DMSO) was made fresh daily and diluted to 240 μg/ml (0.4% DMSO) in pipette solution immediately prior to use. The control bath solution for all experiments was a PSS containing (in mM): 140 NaCl, 5.4 KCl, 1.3 CaCl_2_, 1.0 MgCl_2_, 20 glucose, and 25 4-(2-hydroxyethyl)-1-piperazineethanesulfonic acid (HEPES) (pH to 7.4 with NaOH). The pipette solution consisted of (in mM): 75 K_2_SO_4_, 55 KCl, 5 MgSO_4_, and 10 HEPES (titrated to pH 7.2 with *N*-methyl-D-glucamine).

Membrane potentials were recorded for 200 s. The mean and standard deviation of each record were determined for the entire 200 s. Resting membrane potentials were determined as the means of voltage traces in the absence of action potentials or excitatory post-synaptic potentials. Input resistances were calculated using measured membrane potential depolarizations in response to 0.1 nA current injections (R_input_ = ΔV_m_/I_inject_).

### Neuronal Metabolism Assay

Metabolism in neurons was measured using the resazurin-resorufin assay whereby dark blue redox dye (resazurin) is oxidized into a fluorescent/pink end product (resorufin). The neuronal cells isolated as indicated above were cultured in 12 well plates and treated with normal media or media containing either 4 mM azide or 4 mM azide with 100 μM afobazole for 24 h. After the 24 h incubation, the solutions were removed and replaced with normal culture media. For the assay, 20 μl CellTiter-Blue^TM^ Reagent was added per 100 μl of culture medium in each well. Plates were gently shaken and incubated under standard cell culture conditions for 2 h. Plates were then analyzed using an absorbance reader at 570 and 600 nm.

### Compounds, Reagents, and Antibodies

The following compounds and reagents were used in this investigation: DTG (Tocris Biosciences, Ellisville, MO, United States); Live/Dead Viability/Cytotoxicity Kit (Life Technologies); Sodium Azide (ACROS Organics, Fair Lawn, NJ, United States); Fura-2AM (Molecular Probes, Eugene, OR, United States). The following antibodies were also used: Alexa Fluor 488 anti-mouse IgG (A-11001) and anti-rabbit IgG (A-11008) (Life Technologies); anti-Bax (ab5714), anti-activated caspase-3 (ab32351) and anti-Bcl-2 (ab32370) (Abcam, Cambridge, MA, United States). Afobazole was generously provided by IBC Generium (Moscow, Russia). The vehicle for drugs and reagents used were either water (H_2_O), ethanol (EtOH) or dimethyl sulfoxide (DMSO), and appropriate vehicle controls were carried out for each study.

### Data Analysis

Data were analyzed using a Student’s *t*-test, one-way ANOVA or two-way ANOVA, as appropriate. ANOVAs indicating significant difference were followed with *post hoc* analysis using Holm-Sidak method for determining significant differences between and within individual groups. Results were considered statistically significant if *p* < 0.05.

## Results

### Microglial Activation and Survival

**FIGURE 1:**
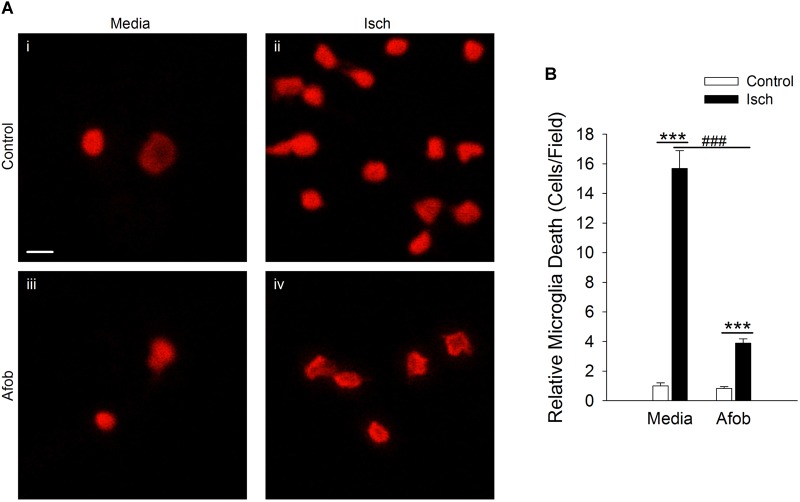
Afobazole reduces microglia death induced by ischemia. **(A)** Photomicrographs showing EthD-1 labeling (red) of microglial cells following 72 h incubation in media alone **(i)**, or media containing 4 mM Azide [*in vitro* ischemia (Isch)] **(ii)**, 30 μM afobazole (Afob) **(iii)**, or 30 μM afobazole + 4 mM Azide **(iv)**. Fields of view shown are representative regions within larger images used to calculate cell death. **(B)** Bar graph of relative cell death observed following 72 h incubation of microglia in control (Control) or ischemic conditions (Isch), with (Afob) or without (Media) 30 μM afobazole. Data were normalized to cell death observed with media alone (Control) (*n* > 12) Scale bar in **(i)** is 10 μm. Asterisks indicate significant difference between Control and Isch within Media and Afob, respectively (*p* < 0.001), and pound symbols denote significant difference between Media and Afob within Isch (*p* < 0.001).

Experiments in our laboratory have shown that σ receptor activation with afobazole can reduce activation of microglia and lessen microglial cell death *in vitro* and *in vivo* (Cuevas et al., 2011b; Katnik et al., 2016). Previous studies have shown that following an ischemic stroke there is an increase in ATP release, which leads to activation and migration of microglial cells (Davalos et al., 2005). Our laboratory has previously shown that afobazole can decrease microglial migration in response to ATP application, but effects on membrane ruffling have not been examined (Cuevas et al., 2011a,b). Treatment with afobazole showed a decrease in microglial activation as reflected in the degree of membrane ruffling in response to ATP (Supplementary Figure S1). Next, a cell death cytotoxicity assay was used to examine the effects of σ receptor activation on the survival of microglia in response to ischemia. Ethidium Homodimer-1 (EthD-1) labeled microglia were counted in the absence (Control) and presence of ischemia (Isch). Incubation for 72 h in media alone (Figure 1Ai,B) or media with 30 μM afobazole (Figure 1Aiii,B) had minimal effect on microglia death, respectively. However, there was significant microglia death following 72 h ischemic incubation (Figure 1Aii,B). Application of afobazole (Afob) significantly mitigated microglial cell death by 75 ± 8% when co-incubated with ischemia (Figure 1Aiv,B).

### Regulation of Pro- and Anti-apoptotic Proteins in Microglia

**FIGURE 2:**
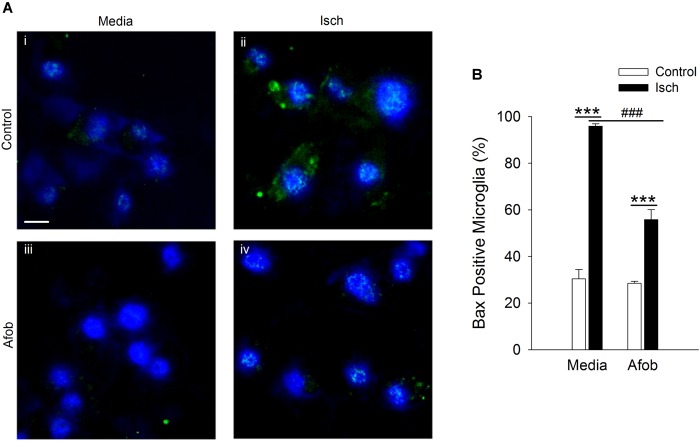
Afobazole prevents upregulation of the pro-apoptotic gene product, Bax, observed in response to ischemia in microglia. Microglia were double labeled with DAPI (blue) and anti-Bax antibody (Alexa Fluor 488 conjugated secondary antibody, green). **(A)** Photomicrographs of merged images of cultured microglia exposed for 24 h to media alone **(i)** or media containing 4 mM Azide (Isch) **(ii)**, 30 μM afobazole **(iii)**, or 4 mM Azide + 30 μM afobazole **(iv)**. Fields of view shown are representative regions within larger images used to calculate number of Bax-positive cells. Scale bar in **(i)** is 10 μm. **(B)** Bar graph of mean number of Bax-positive microglia in experiments in which the cells were exposed to normal conditions (Control) or ischemia (Isch) in the absence (Media) or presence of 30 μM afobazole (Afob) (*n* = 9). Asterisks indicate significant difference between Control and Isch within Media and Afob, respectively (*p* < 0.001), and pound symbols denote significant difference between Media and Afob within Isch (*p* < 0.001).

The mechanism(s) by which activation of σ receptors decreases cell death in microglia remain unknown. Thus, experiments were carried out to determine if activation of σ receptors alters the expression of proteins involved in cell apoptosis in microglia. One pro-apoptotic gene product that has been shown to be affected by ischemia and may be regulated by σ receptors is the protein Bax (Schelman et al., 2004; Tchedre and Yorio, 2008). Immunocytochemical experiments were conducted to measure Bax expression in microglia following a 24 h incubation under ischemic conditions to ascertain whether afobazole application affects expression of this protein. Imaging of microglia showed that Bax labeling was rarely observed in control cells (Figure 2ii) or cells exposed to afobazole alone (Figure 2iii). Bax labeling was readily observed after ischemia (Figure 2Aii), but was not as prominent under control conditions (Figure 2Ai) or when these cells were incubated in afobazole during the ischemia (Figure 2Aiv). Co-labeling of microglia with DAPI and an anti-Bax antibody shows that Bax is detected in few control cells but is highly expressed following ischemia (Figure 2B). Afobazole alone had no effect on endogenous Bax levels but significantly reduced by 40 ± 5% the number of microglia expressing Bax after ischemia (Figure 2B).

**FIGURE 3:**
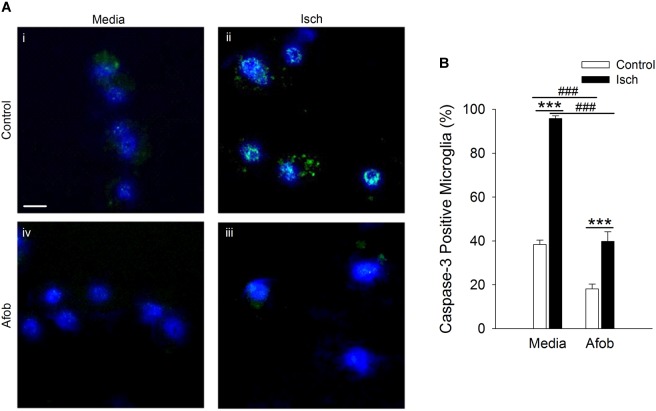
Afobazole reduces expression of caspase-3 in response to ischemia in microglia. Microglia were double labeled with DAPI (blue) and anti-caspase-3 antibody (Alexa Fluor 488 conjugated secondary antibody, green). **(A)** Photomicrographs of merged images of cultured microglia exposed for 24 h to media alone **(i)** or media containing 4 mM Azide (Isch) **(ii)**, 30 μM afobazole **(iii)**, or 4 mM Azide + 30 μM afobazole **(iv)**. Fields of view shown are representative regions within larger images used to calculate caspase-3 expression levels. Scale bar in **(i)** is 10 μm. **(B)** Bar graph of mean percentage of caspase-3-positive microglia in experiments in which the cells were exposed to normal conditions (Control) or ischemia (Isch) using media alone (Media) or media with 30 μM afobazole (Afob) (*n* = 9). Asterisks indicate significant difference between Control and Isch within Media and Afob, respectively (*p* < 0.001), and pound symbols denote significant difference between Media and Afob within Control and Isch, respectively (*p* < 0.001).

Bax-dependent caspase-3 activation is a key component in cellular apoptosis (Cregan et al., 1999). Therefore, it was examined how afobazole effects the levels of the death protease, caspase-3, in microglia after ischemia treatment. Figure 3A shows photomicrographs of microglia double labeled with DAPI (blue) and for caspase-3 (green). There was a notable increase in the number of microglia expressing caspase-3 following ischemia (Figure 3Ai,ii) and this upregulation was reduced by afobazole treatment (Figure 3Aiii,iv). Quantification of the images showed that afobazole alone significantly decreased caspase-3 expressing cells by 20 ± 3% relative to the control and diminished the increases in caspase-3 evoked by ischemia by 56 ± 5% (Figure 3B).

**FIGURE 4:**
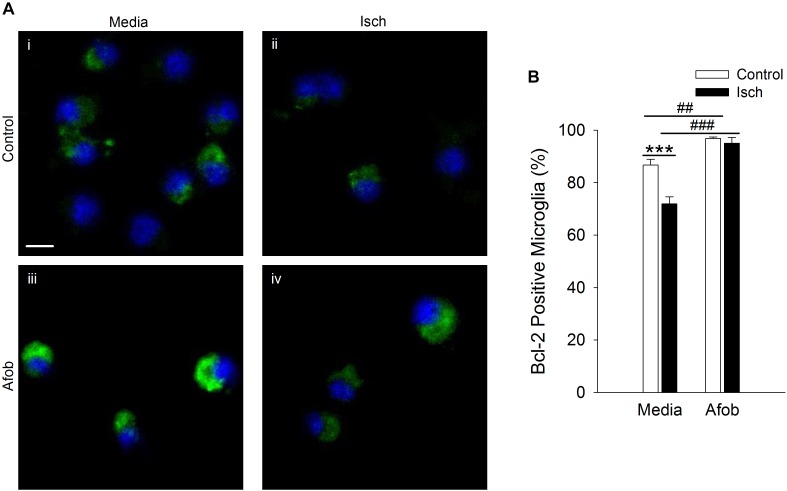
Afobazole enhances Bcl-2 expression under control conditions and following ischemia in microglia. Microglia were double labeled with DAPI (blue) and anti-Bcl-2 antibody (Alexa Fluor 488 conjugated secondary antibody, green). **(A)** Photomicrographs of merged images of cultured microglia exposed for 24 h to media alone **(i)** or media containing 4 mM Azide **(ii)**, 30 μM afobazole **(iii)** or 4 mM Azide + 30 μM afobazole **(iv)**. Areas shown are representative regions within larger images used to calculate Bcl-2 expression. Scale bar in **(i)** is 10 μm. **(B)** Bar graph of mean percentage of Bcl-2-positive microglia in experiments in which the cells were exposed to normal conditions (Control) or ischemia (Isch), in the absence (Media) and presence of 30 μM afobazole (Afob) (*n* = 9). Asterisks indicate significant difference between Control and Isch within Media (*p* < 0.001), and pound symbols denote significant difference between Media and Afob within Control and Isch, respectively (*p* < 0.001).

The effects of Bax can be counteracted by upregulation of the anti-apoptotic protein Bcl-2 (Hockenbery et al., 1993; Korsmeyer et al., 1993; Oltvai et al., 1993), and this may be a second mechanism by which σ receptors reduce cell death following ischemia. The relationship between afobazole and the expression of the anti-apoptotic gene, Bcl-2, was examined using immunocytochemical analysis of microglia following ischemia. Figure 4A shows photomicrographs of microglia double labeled with DAPI (blue) and for Bcl-2 (green). The majority of microglia were found to constitutively express Bcl-2 under control conditions (Figure 4Ai), and this protein was detected both following ischemia (Figure 4Aii) and when cells were incubated in afobazole (Figure 4Aiii). Ischemia reduced the number of microglia expressing Bcl-2 by 15 ± 3% relative to control (Figure 4B). Afobazole alone produced a 10 ± 2% increase in the number of cells expressing Bcl-2. Moreover, afobazole rescued Bcl-2 expression following ischemia, with nearly 100% of cells expressing this anti-apoptotic protein under both control and ischemic conditions.

### Neuronal Cell Death and Regulation of Bax, Caspase-3, and Bcl-2

**FIGURE 5:**
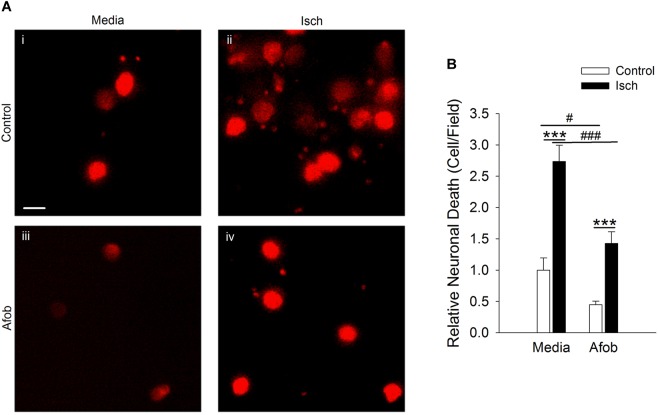
Afobazole reduces neuronal death induced by ischemic conditions. Neurons labeled with EthD-1 (red). **(A)** Photomicrographs of images of cultured neurons exposed for 72 h to media alone **(i)** or media containing 4 mM Azide **(ii)**, 30 μM afobazole **(iii)**, or 4 mM Azide + 30 μM afobazole **(iv)**. Areas shown are representative regions within larger images used to calculate cell death. Scale bar in **(i)** is 10 μm. **(B)** Bar graph of relative cell death observed following 72 h incubation of neurons in media (Media) or media containing ischemia (Isch), 100 μM afobazole (Afob) or ischemia + 100 μM afobazole (Isch + Afob). Data were normalized to cell death observed with media alone (Control) (*n* > 12). Asterisks indicate significant difference between Control and Isch within Media and Afob, respectively (*p* < 0.001), and pound symbols denote significant difference between Media and Afob within Control and Isch, respectively (#*p* < 0.05; ###*p* < 0.001).

Previous studies revealed that σ receptor activation decreases neuronal death following a 1 h ischemic event *in vitro* (Cuevas et al., 2011a). However, it has not been determined if neuroprotection occurs following longer ischemic events such as those used with microglia in this study. Thus, experiments were carried out to see if afobazole can decrease neuronal death under these conditions. Neurons labeled with EthD-1 were counted following a 72 h incubation under control conditions (Control) and under ischemic conditions (Isch). Figure 5A shows photomicrographs of microglia labeled with EthD-1 (red). There is a notable increase in neurons positive for EthD-1 following ischemia (Figure 5Ai,ii) and a reduction when afobazole was applied (Figure 5Aiii,iv). Counting the number of EthD-1 positive cells under the different conditions indicates that there was significant 173 ± 27% increase in neuronal death in response to ischemia, relative to control (Figure 5B). When σ receptors were activated with afobazole, the neuronal death caused by ischemia was reduced by 131 ± 29% (Figure 5B).

**FIGURE 6:**
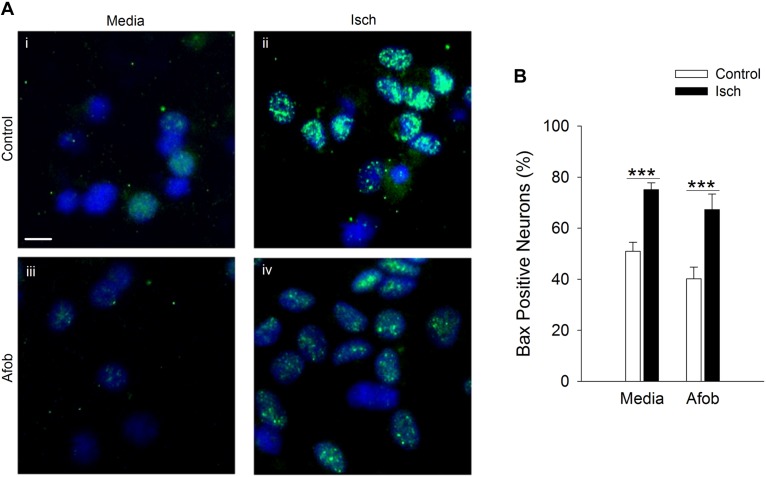
Afobazole fails to prevent upregulation of the pro-apoptotic gene product, Bax, observed in response to extended ischemia in neurons. **(A)** Photomicrographs of merged images of cultured neurons exposed for 24 h to media alone **(i)**, or media containing 4 mM Azide **(ii)**, 100 μM afobazole (Afob) **(iii)** or 4 mM Azide + 100 μM afobazole (Isch + Afob) **(iv)**. Neurons were double labeled with DAPI (blue) and anti-Bax antibody (green). Fields of view shown are representative regions within larger images used to calculate Bax expression. Scale bar in **(i)** is 10 μm. **(B)** Bar graph of mean percentage of Bax-positive neurons in experiments in which the cells were exposed to the same conditions as **(A)** (*n* = 9). Asterisks indicate significant difference between Control and Isch within Media and Afob, respectively (*p* < 0.001).

Experiments were next carried out to determine if the enhanced neuronal survival observed following σ receptor stimulation in neurons involves a pathway similar to that observed in microglial cells. As in microglia, Bax labeling was readily visible in neurons (Figure 6Ai,iii) and appeared to be increased by ischemia (Figure 6Aii,iv). Exposing neurons to afobazole under control conditions produced a slight reduction in the number of cells expressing Bax, but this was not significant (Figure 6B). Similarly, when afobazole was applied during ischemia, Bax expression was reduced by ∼10% compared to ischemia alone, but this reduction was not significant (Figure 6B). Thus, unlike the case in microglia, σ receptor activation fails to significantly block ischemia-induced upregulation of the pro-apoptotic protein, Bax, in neurons.

**FIGURE 7:**
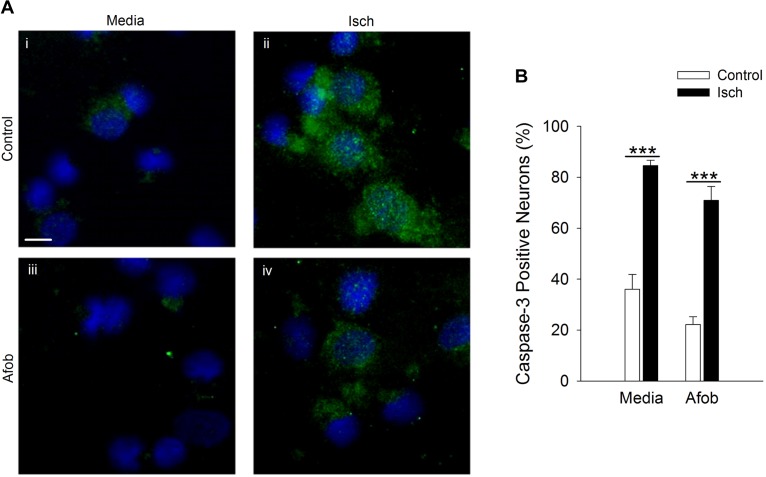
Afobazole fails to reduce expression of caspase-3 in response to prolonged ischemia in neurons. **(A)** Photomicrographs of merged images of cultured neurons exposed for 24 h to media alone (Control) **(i)**, or media containing 4 mM Azide (Isch) **(ii)**, 100 μM afobazole (Afob) **(iii)** or 4 mM Azide + 100 μM afobazole (Isch + Afob) **(iv)**. Neurons were double labeled with DAPI (blue) and anti-active caspase-3 antibody (green). Fields of view of each photomicrograph are representative regions within larger image used to calculate caspase-3 expression. Scale bar in **(i)** is 10 μm. **(B)** Bar graph of mean percent of caspase-3-positive neurons in experiments in which the cells were exposed to conditions identical to **(A)** (*n* = 9). Asterisks indicate significant difference between Control and Isch within Media and Afob, respectively (*p* < 0.001).

It was further examined whether afobazole effects the levels of the death protease, caspase-3, in neurons after an ischemic insult. Following ischemia, caspase-3 expression was observed in a greater number of cells relative to control in the absence and presence of afobazole (Figure 7A). Examining the number of cells staining positive for caspase-3 demonstrates that afobazole reduces the number of caspase-3 expressing cells by 14 ± 5% in the absence of ischemia, but this difference was not statistically significant (Figure 7B). As with Bax, afobazole reduced caspase-3 levels following ischemia, but this effect was also not statistically significant (Figure 7B).

**FIGURE 8:**
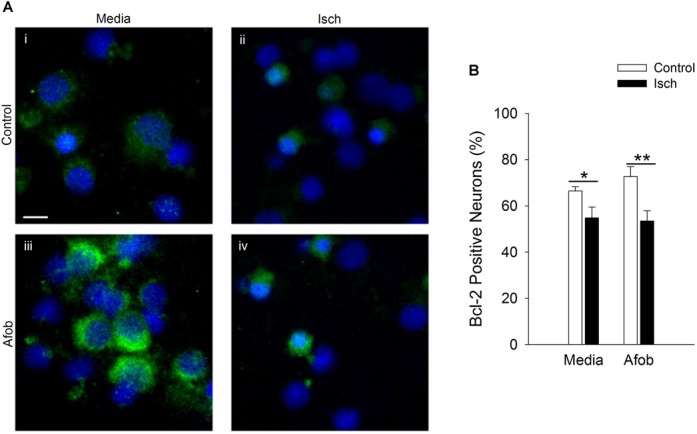
Activation of σ receptors does not upregulate Bcl-2 levels following 24 h ischemia. Neurons were double labeled with DAPI (blue) and anti-Bcl-2 antibody (Alexa Fluor 488 conjugated secondary antibody, green). **(A)** Photomicrographs of merged images of cultured neurons exposed for 24 h to media alone **(i)** or media containing 4 mM Azide (**ii**, Isch), 100 μM afobazole **(iii)** or 4 mM Azide + 100 μM afobazole **(iv)**. Areas shown are representative regions within larger images used to calculate Bcl-2 expression levels. Scale bar in **(i)** is 10 μm. **(B)** Bar graph of mean percent of Bcl-2-positive neurons in experiments in which the cells were exposed to conditions identical to **(A)** (*n* = 9). Asterisks indicate significant difference between Control and Isch within Media and Afob, respectively (^∗^*p* < 0.05; ^∗∗^*p* < 0.01).

Given that activation of σ receptors failed to lessen the pro-apoptotic gene burden in neurons following ischemia, experiments were carried out to determine if σ receptors affected anti-apoptotic gene expression as a means by which to decrease ischemia-induced cell death. As with microglia, Bcl-2 labeling was readily visible under all conditions tested (Figure 8A). Following ischemia, the number of neurons expressing the anti-apoptotic protein, Bcl-2, significantly decreased by 12 ± 5 and 19 ± 8% in cells exposed to ischemia alone or ischemia in the presence of afobazole, respectively (Figure 8B). Thus, afobazole failed to significantly blunt the reduction in Bcl-2 produced by ischemia. The failure of σ receptors to affect Bax, caspase-3, and Bcl-2 levels in neurons following ischemia suggests that different pathways must be involved in σ receptor protection of neurons and microglia, respectively.

### Functional Properties of Neurons Post-ischemia

**FIGURE 9:**
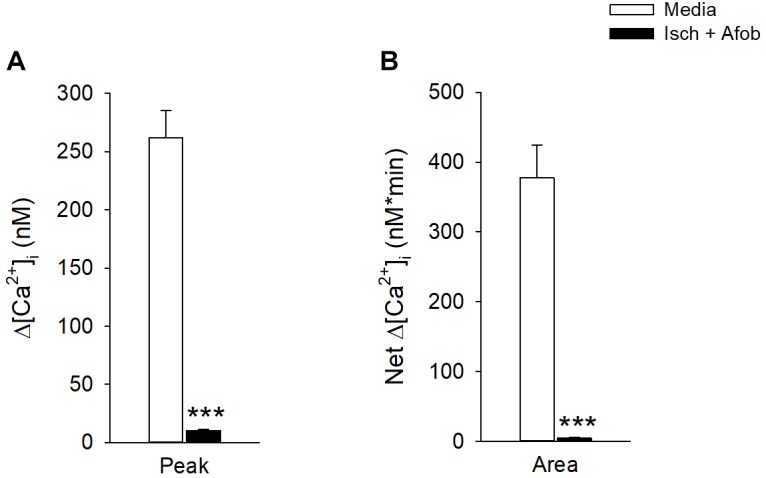
Afobazole fails to preserve neuronal membrane responses to elevated external K^+^ after prolonged ischemia. Bar graphs of mean change in depolarization-induced increase in Peak [Ca^2+^]_i_ **(A)** and total net calcium influx (Area) **(B)** evoked by application of high K^+^ (30 mM KCl) in isolated cortical neurons. Neurons were incubated for 24 h with afobazole alone (Media) or under ischemic conditions with 100 μM afobazole (Isch + Afob). Ischemic solution and afobazole were removed and neurons then incubated in normal media during a 72 h recovery (*n* ≥ 79). Asterisks indicate significant difference between Media and Isch + Afob (*p* < 0.001).

While σ receptor activation produced a reduction in neuronal apoptosis in response to ischemia, the functional status of surviving neurons is unknown. A previous study in our laboratory showed that activation of σ receptors does preserve microglial function after ischemia (Cuevas et al., 2011b). Thus, experiments were carried out to determine if the surviving neurons had preserved functional properties like microglia, or if neuronal activity was compromised. Fluorometric calcium imaging experiments were conducted to determine if surviving cortical neurons showed responses to membrane depolarization. Neurons were incubated for 24 h under ischemic conditions in the presence of 100 μM afobazole and were then permitted to recover for 72 h in normal media. As a control, we incubated neurons for 24 h in the presence of afobazole, followed by a 72 h incubation in media alone. Following the recovery period, neurons were stimulated by focal, transient application of 30 mM KCl. In contrast to control neurons (Media), neurons incubated under ischemic conditions in the presence of afobazole (Isch+Afob) failed to respond to high-K^+^ application (Figure 9). Both peak increases in [Ca^2+^]_i_ (Figure 9A) and net increases in [Ca^2+^]_i_ (Figure 9B) were significantly reduced by ischemia with or without afobazole incubation. This observation indicates that voltage-gated Ca^2+^ channels are not being activated in these cells in response to predicted membrane depolarizations following ischemia. This could be either due to steady-state inactivation, functional depression or decreased expression of the voltage-gated Ca^2+^ channels. Alternatively, changes in the K^+^ channels that set resting membrane potentials and depolarize the neurons in the presence of elevated external K^+^ concentrations could be altered. These data suggest that while afobazole reduced neuronal cell death, the surviving neurons are functionally compromised even after 72 h recovery.

**FIGURE 10:**
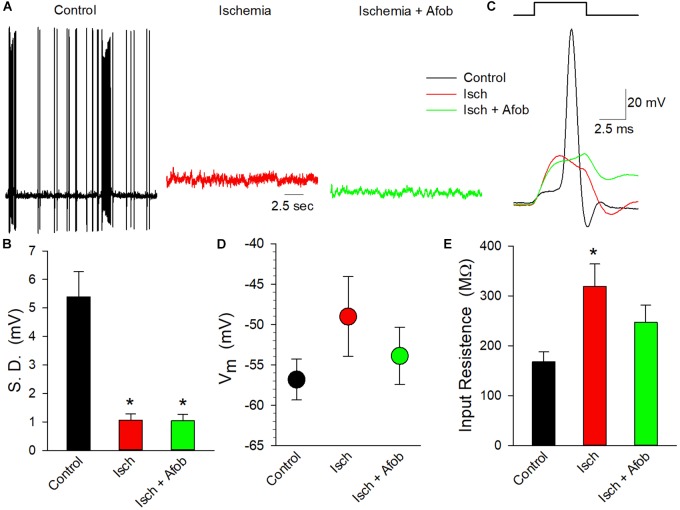
Afobazole fails to preserve electrical activity in cortical neurons following ischemia. **(A)** Traces of electrical activity recorded from three different neurons following incubation under normal conditions (Control, black trace), under ischemic conditions (Isch, red trace) and under ischemic conditions in the presence of 100 μM afobazole (Isch + Afob, green trace). Neurons were incubated for 24 h under ischemic conditions ( ± afobazole) and permitted to recover for 72 h. **(B)** Mean ( ± SEM) of the standard deviations of the membrane potentials recorded from individual neurons under the same conditions as **(A)** (Control, *n* = 10; Isch, *n* = 16; Isch + Afob, *n* = 13). **(C)** Representative traces of membrane responses recorded following a 0.1 nA current injection from neurons under the same conditions as **(A)**. Resting membrane potential **(D)** and input resistance **(E)** recorded from same neurons as **(B)**. Asterisks in **(B,E)** denote significant difference from Control (*p* < 0.05).

To determine if the electrical activity of these neurons had been altered by ischemia in a manner not affected by incubation in afobazole, whole-cell patch-clamp recordings were carried out on isolated cortical neurons. Cells were studied after incubation under normal conditions or after 24 h of ischemia in the absence or presence of 100 μM afobazole, followed by a 72 h recovery in normal media. In membrane potential recordings from cells incubated under normal conditions (Control) spontaneous actions potentials were readily observed, and bursting behavior was common (Figure 10A, black trace). In contrast, incubation of the cells under ischemic conditions, without (Isch) or with (Isch + Afob) afobazole, resulted in a decrease in observed spontaneous activity (Figure 10A, red and green traces). The degree of membrane activity was quantified by determining the standard deviation of the membrane potential from the mean membrane potential. Following ischemia, the electrical activity in the neurons was significantly decreased, and incubation with afobazole during ischemia failed to blunt this effect (Figure 10B). In addition to this decrease in spontaneous activity, application of depolarizing current pulses (100 pA) always evoked action potentials in Control neurons but failed to in any of the neurons exposed to ischemia or ischemia in the presence of afobazole (Figure 10C). While there was a trend toward a more positive resting membrane potential in neurons exposed to ischemia alone relative to control and afobazole groups, there was not a statistically significant effect of ischemia on surviving neurons (Figure 10D). In contrast, input resistance, which indicates ion channel activity and membrane electrical permeability, was significantly increased in the ischemia group relative to control (Figure 10E). Incubation with afobazole during the ischemic event reduced input resistance such that these cells were no longer statistically different from control (Figure 10E). This is suggestive of reduced K^+^ channel activity following ischemia that is lessened by σ receptor application.

**FIGURE 11:**
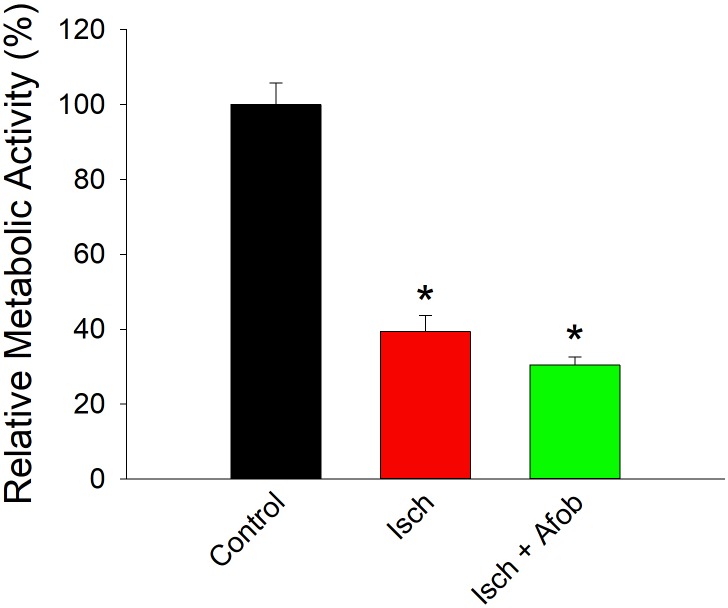
Afobazole fails to prevent reduced metabolic activity in neurons caused by ischemia. Bar graph of relative metabolic activity as determined with the resazurin-resorufin fluorescence assay. Values obtained for individual cultures incubated under ischemic conditions in the absence (Isch) and presence (Isch + Afob) of 100 μM afobazole are expressed as a percent of the average value obtained from cultures incubated in normal media (Control) (*n* = 4 for all groups). Asterisk denote significant difference from Control (*p* < 0.001 for both).

Given the reduction in electrical activity, experiments were carried out to determine how ischemia affected the metabolic activity in surviving neurons and if afobazole affected these properties. The metabolic activity of isolated cortical neurons was determined using the resazurin-resorufin fluorescence assay. Figure 11 shows a bar graph of metabolic activity in neurons expressed as a percent of average activity measured in control neurons. Following ischemia (24 h) and recovery (72 h), metabolic activity was significantly depressed in surviving neurons (Isch, red bar) relative to control (Control, black bar). Similarly, neurons incubated with afobazole (100 μM) during the ischemic event also had significantly reduced metabolic activity (Isch+Afob, green bar) compared to control. Taken together, these data further support the conclusion that afobazole fails to preserve the functional integrity of neurons exposed to ischemia.

## Discussion

The most prominent finding of this study is that the σ receptor agonist, afobazole, rescues microglia, but not neurons, from ischemia-induced cell death by regulating the expression of the pro-apoptotic proteins, Bax and caspase-3, and the anti-apoptotic protein Bcl-2. Microglia treated with afobazole during long-term ischemia (≥24 h) were less likely to express both the pro-apoptotic gene product, Bax, and the death protease, caspase-3. Furthermore, afobazole treatment resulted in an upregulation of the anti-apoptotic gene product, Bcl-2, in microglia such that a significantly greater number of cells were found to express this protein. Afobazole was also found to reduce cell death in cortical neurons, but surprisingly, the σ receptor agonist failed to alter the expression of these three proteins in a manner consistent with an anti-apoptotic effect. In addition, activation of σ receptors failed to preserve the functional properties in neurons following ischemia, in contrast to what has been reported for microglia previously (Cuevas et al., 2011b). These findings suggest that activation of σ receptors differentially affects survival mechanisms in neurons and microglia and the functional resilience of these cells following ischemia.

Previously studies have shown that σ receptor activation can mitigate intracellular calcium dyshomeostasis in neurons and microglia following ischemia, acidosis, ATP and amyloid-β exposure (Katnik et al., 2006; Herrera et al., 2008; Hall et al., 2009a; Cuevas et al., 2011a,b; Behensky et al., 2013a,b,c; Mari et al., 2015). Sustained elevations in intracellular calcium are associated with the initiation of apoptosis (Mattson and Chan, 2003), and thus by preserving intracellular calcium homeostasis, afobazole may prevent cell death. Findings reported here demonstrate that afobazole also reduces microglial, but not neuronal, cell death by decreasing Bax expression following ischemic stroke. Afobazole has been previously shown to decrease Bax expression in microglia and neurons following exposure to amyloid-β_25-35_ (Behensky et al., 2013b,c). Similarly, activation of σ-1 receptors with ANAVEX2-73 has been shown to decrease Bax expression in the mouse hippocampus in response to amyloid-β_25-35_ (Lahmy et al., 2014). In contrast to these earlier studies, but consistent with observations reported here, the σ-1 receptor agonist, 4-phenyl-1-(4-phenylbutyl) piperidine (PPBP) increases neuronal survival in response to ischemia via a Bax-independent mechanism (Yang et al., 2007). While most studies have reported either a decrease in Bax or no changes in this protein in response to σ receptor stimulation, microglial activation and death caused by methamphetamine appears to be due to methamphetamine stimulation of σ-1 receptors and upregulation of Bax (Shen et al., 2016). Thus, the effects of σ receptor activation on Bax levels seems to be cell and agonist dependent, and is variable depending on the CNS injury model being examined (i.e., ischemia vs. amyloid-β_25-35_ toxicity).

Activation of caspase-3 downstream of Bax has been shown to be an important contributing factor to cell death following cerebral ischemia (Liu et al., 2013). The current study demonstrates that activation of σ receptors with afobazole reduces upregulation of activated caspase-3 in response to ischemia in microglial cell, but that this upregulation is still present in neurons. Afobazole activation of σ receptors was previously shown to lessen activated caspase-3 burden in microglial and neuronal cells in an amyloid-β_25-35_ toxicity model (Behensky et al., 2013b,c). Thus, σ receptor-mediated cytoprotection triggered by afobazole is in part due to depression of caspase-3 activation in microglial cells, but not neurons. The results for ischemic microglial cells parallel those observed for afobazole inhibition of caspase-3 activation following amyloid-β_25-35_ toxicity, but differ for results obtained with neurons in the Aβ model (Behensky et al., 2013b,c). Activation of σ receptors with the σ-1 agonist, (+) pentazocine, has been shown to decrease caspase-3 upregulation caused by ischemia in retinal ganglion cells (Ellis et al., 2017). In the hyperoxia model of developing brain injury the σ-1 agonist, dextromethorphan, but not the σ-1 agonist, PRE-084, decreased caspase-3 activation in both neurons and glial cells (Posod et al., 2013, 2014). As with Bax, caspase-3 was also activated in microglial cells by methamphetamine stimulation of the σ-1 receptor (Shen et al., 2016). Thus, caspase-3 has a complex relationship with the σ-1 receptor and modulation of this pro-apoptotic protein by σ-1 receptors is dependent on ligand, cell type, and injury model studied. To date, the effects of σ-2 receptor stimulation on caspase-3 have been studied exclusively on σ-2 receptors in tumor cells and studies indicate a correlation between σ-2 stimulation and caspase-3 activation (Pati et al., 2017). The effects of selective σ-2 receptor stimulation on caspase-3 activation remain to be determined in neurons and microglia. However, data from our laboratory suggest that activation of σ-2 contributes to increased microglial cell and neuron survival after ischemia, and thus, σ-2 is unlikely to upregulate pro-apoptotic pathways in these cells (Cuevas et al., 2011a,b; Katnik et al., 2014, 2016).

In addition to inhibiting increases in Bax and activated caspase-3 in microglia, afobazole produced an increase of Bcl-2 expression in microglia that was more pronounced following ischemia. Similar increases in microglial expression of Bcl-2 due to σ receptor stimulation with afobazole were observed previously in cells exposed to amyloid-β_25-35_ toxicity (Behensky et al., 2013c). However, afobazole failed to prevent downregulation of Bcl-2 in neurons in response to ischemia in the current study, but was previously shown to inhibit amyloid-β_25-35_ toxicity-evoked depression of Bcl-2 expression (Behensky et al., 2013b). Therefore, there is overlap in the mechanisms by which afobazole activation of σ receptors prevents apoptotic cell death in microglia caused by amyloid-β_25-35_ toxicity and ischemia. In contrast, the mechanisms responsible for afobazole reduction of ischemia-induced cell death in neurons and microglia differ. The σ-1 agonist, ANAVEX2-73, has also been shown to protect neurons from amyloid-β_25-35_ toxicity, but this effect was not dependent on upregulation of Bcl-2 (Lahmy et al., 2014). In contrast to afobazole, the σ-1 agonist, PPBP, was previously shown to decrease cell death caused by oxygen-glucose deprivation in neurons via upregulation of Bcl-2 (Yang et al., 2007). Thus, as with Bax and activated caspase-3, the effects of σ receptor agonists on Bcl-2 vary by drug and disease state.

Data presented here clearly demonstrate that afobazole decreased neuronal death even when cells were exposed to ischemic conditions for 72 h. Numerous studies had previously reported that activation of σ receptors is neuroprotective and that afobazole decreases cell death via activation of these receptors following ischemia. However, the role of the Bax/caspase-3/Bcl-2 pathway had not previously been comprehensively tested, particularly with afobazole as the agonist. Surprisingly, neurons surviving the ischemic insult failed to respond to focal application of 30 mM KCl with Ca^2+^ elevations caused by membrane depolarizations, suggesting a functional downregulation or reduced expression of voltage-gated Ca^2+^ channels. Following transient global cerebral ischemia in rats, a downregulation of L-type Ca^2+^ channel currents was observed with no reduction in Ca^2+^ channel protein levels, indicating that channel activity was compromised (Li et al., 2007). It remains to be determined if a similar effect is responsible for the reduced Ca^2+^ influx observed here. In addition, neurons in the current study failed to exhibit normal spontaneous activity or evoked action potentials and had metabolic activities significantly lower than control neurons. Thus, these neurons appear to be alive but functionally compromised. It remains to be determined if this failure to respond is indicative of neurons that will ultimately undergo apoptosis or another other form of cell death, or if this quiescent state is a mechanism for long-term cell survival under stress. For example, it has been shown that brain regions surviving ischemic episodes can recover from isoelectric silence and ultimately exhibit neuronal activity (Wu et al., 2012). However, the inability of afobazole to preserve neuronal function at these later stages post-ischemia suggests that the *in vivo* benefits of sigma receptor activation likely depend on mechanisms beyond neuroprotection for long-term effects.

## Conclusion

Our study demonstrates that afobazole can reduce microglial toxicity and cell death following prolonged ischemic exposure. This cytoprotection may involve regulation of multiple genes, with a noted decrease in Bax and caspase-3 expression levels and a concomitant increase in Bcl-2 expression. Furthermore, our experiments show that afobazole can decrease ischemia-evoked neuronal cell death. However, the molecular mechanisms mediating neuroprotection by afobazole following an extended ischemic insult appear to be different for neurons than with microglia and do not seem to involve the regulation of apoptotic genes. In contrast to the effects on microglia reported previously (Cuevas et al., 2011b), afobazole fails to preserve neuronal function following recovery from ischemia. Taken together our data suggest that afobazole may prevent microglia from entering the activated state associated with neurotoxicity following exposure to ischemia and increases the survival of both microglia and neurons following an ischemic insult. These properties make afobazole an attractive drug for treatment of ischemic stroke.

## Data Availability

The datasets generated for this study are available on request to the corresponding author.

## Ethics Statement

This study was carried out in accordance with the recommendations of the University of South Florida’s Institutional Animal Care and Use Committee. The protocol was approved by the University of South Florida’s Institutional Animal Care and Use Committee.

## Author Contributions

AB, CK, HY, and JC participated in research design, performed the data analysis and wrote or contributed to the writing of the manuscript. AB, CK, and HY conducted the experiments.

## Conflict of Interest Statement

The authors declare that the research was conducted in the absence of any commercial or financial relationships that could be construed as a potential conflict of interest.

## Supplementary Material

FIGURE S1 Afobazole blocks membrane ruffling induced by ATP in microglial cells. **(A)** Photomicrographs of microglial cells, labeled with the filamentous actin probe phalloidin, exposed for 10 min (37°C) to media alone (Control), media containing 30 μM afobazole (Afob), or media containing 30 μM DTG (DTG) in the absence **(i,iii,v)** and presence **(ii,iv,vi)** of 100 μM ATP, respectively. Insets show zoomed in images of the indicated regions. Scale bar = 3 μm. **(B)** Bar graph of mean degree of membrane ruffling observed in cells exposed to the same conditions as **(A)** (*n* > 67). Cell ruffling was scored as follows: “0,” no ruffling and multiple filopodia; “1,” ruffling and filopodia; and “2,” fully ruffled with no filopodia. Asterisks indicate significant difference between Control and ATP within Media (*p* < 0.001), 30 μM afobazole (Afob, *p* < 0.05) and 30 μM DTG (DTG, *p* < 0.01). Pound symbols denote significant difference between Media and sigma agonists (Afob and DTG) within ATP (*p* < 0.001).
