# Supplementary figures and images for: Activation of Sigma Receptors With Afobazole Modulates Microglial, but Not Neuronal, Apoptotic Gene Expression in Response to Long-Term Ischemia Exposure
Source: Front Neurosci. 2019 May 15;13:414. doi: 10.3389/fnins.2019.00414 (PMC6529844; doi:10.3389/fnins.2019.00414)

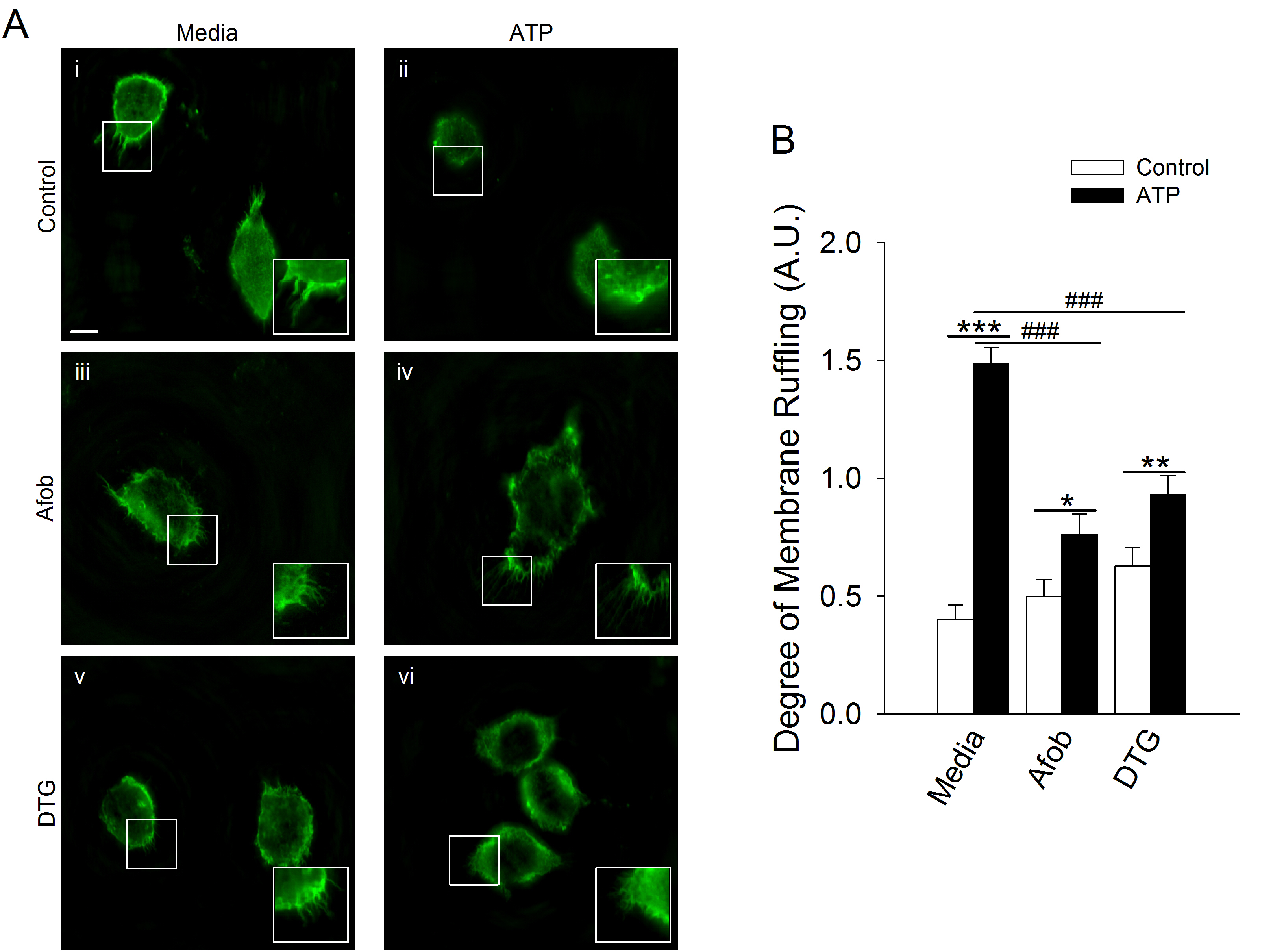

Supplement: FIGURE S1 — Afobazole blocks membrane ruffling induced by ATP in microglial cells. (A) Photomicrographs of microglial cells, labeled with the filamentous actin probe phalloidin, exposed for 10 min (37°C) to media alone (Control), media containing 30 μM afobazole (Afob), or media containing 30 μM DTG (DTG) in the absence (i,iii,v) and presence (ii,iv,vi) of 100 μM ATP, respectively. Insets show zoomed in images of the indicated regions. Scale bar = 3 μm. (B) Bar graph of mean degree of membrane ruffling observed in cells exposed to the same conditions as (A) (n > 67). Cell ruffling was scored as follows: “0,” no ruffling and multiple filopodia; “1,” ruffling and filopodia; and “2,” fully ruffled with no filopodia. Asterisks indicate significant difference between Control and ATP within Media (p < 0.001), 30 μM afobazole (Afob, p < 0.05) and 30 μM DTG (DTG, p < 0.01). Pound symbols denote significant difference between Media and sigma agonists (Afob and DTG) within ATP (p < 0.001). [file Image_1.JPEG]
